# Diagnostic, Theranostic and Prognostic Value of Thyroglobulin in Thyroid Cancer

**DOI:** 10.3390/jcm13092463

**Published:** 2024-04-23

**Authors:** Luca Giovanella, Federica D’Aurizio, Petra Petranović Ovčariček, Rainer Görges

**Affiliations:** 1 Department of Nuclear Medicine, Gruppo Ospedaliero Moncucco SA, Clinica Moncucco, 6900 Lugano, Switzerland; 2 Clinic for Nuclear Medicine, University Hospital and University of Zurich, 8006 Zurich, Switzerland; 3 Institute of Clinical Pathology, Department of Laboratory Medicine, University Hospital of Udine, 33100 Udine, Italy; federica.daurizio@asufc.sanita.fvg.it; 4 Department of Oncology and Nuclear Medicine, University Hospital Center Sestre Milosrdnice, 10000 Zagreb, Croatia; p.petranovic@gmail.com; 5 School of Medicine, University of Zagreb, 10000 Zagreb, Croatia; 6 Clinic for Nuclear Medicine, University Hospital of Essen, 45147 Essen, Germany; rainer.goerges@uni-due.de

**Keywords:** thyroglobulin, differentiated thyroid cancer, diagnostics, prognosis, theranostics, radioiodine

## Abstract

Thyroglobulin (Tg) is an iodinated glycoprotein, which is normally stored in the follicular colloid of the thyroid, being a substrate for thyroid hormone production. Since it is produced by well-differentiated thyroid cells, it is considered a reliable tumor marker for patients with differentiated thyroid carcinoma (DTC) during their follow-up after total thyroidectomy and radioiodine ablation. It is used to monitor residual disease and to detect recurrent disease. After total thyroid ablation, unstimulated highly sensitive Tg measurements are sufficiently accurate to avoid exogenous or endogenous thyrotropin (TSH) stimulation and provide accurate diagnostic and prognostic information in the great majority of DTC patients. Adopting sophisticated statistical analysis, i.e., decision tree models, the use of Tg before radioiodine theranostic administration was demonstrated to be useful in refining conventional, pathology-based risk stratification and providing personalized adjuvant or therapeutic radioiodine administrations. The follow-up of DTC patients aims to promptly identify patients with residual or recurrent disease following primary treatment. Our review paper covers the diagnostic, theranostic and prognostic value of thyroglobulin in DTC patients.

## 1. Introduction

Differentiated thyroid carcinomas, i.e., papillary (PTC) and follicular thyroid carcinomas (FTC), account for the majority of endocrine cancers. Their incidence has increased significantly over the past decades, largely (but not totally) due to the increased use of imaging techniques, mainly ultrasound (US), in clinical practice. More women than men are affected by DTC. Moreover, thyroid cancer is more frequent in women under 25 years of age and in those aged between 45 and 60 years [1]. Luckily, DTC has a slow and non-aggressive course in most cases and, consequently, the prognosis is generally favorable. In any case, at least 10% of cases carry distant metastases at presentation with an attached increase in cancer-related mortality. Additionally, recurrent disease occurs over time in 20–30% with a 10-year mortality rate of 4–7% [2,3].

Multiple biomolecular mechanisms impact the behavior of DTC cells, leading to different phenotypes and prognosis, respectively. The BRAF V600E mutation, as an example, lowers the expression of genes regulating the metabolism of iodine, and in turn, the tumors’ responsiveness to iodine-131 (131I) [4].

Taking into account different molecular and clinical phenotypes, the approach to DTC patients has shifted from a “one size fits all” (i.e., total thyroidectomy plus 131I) to a tailored risk-adapted treatment based on individual risk profile [2,3,5,6].

Since 2015, the ATA risk classification has been largely used to guide 131I therapy, and post-operative 131I administration is proposed in patients at intermediate and high-risk, while it is restricted to selected low-risk cases (e.g., additional risk factors, patient’s preference) [2] (Table 1).

However, the goal of 131I administration (Table 2) can only be determined once the post-operative disease status has been assessed.

In fact, “treatment of known disease” is required in all patients with biochemical, structural or functional evidence of persistent disease, independently of the ATA risk class. Patients with no evidence of disease after initial surgical treatment may undergo observation, remnant ablation or adjuvant therapy depending on their risk profile. After thyroidectomy, levothyroxine is started at 1.5–1.8 μg per kg of body weight and tittered to obtain TSH levels < 0.1 mUI/L in high-risk and 0.1–0.5 mUI/L in low–intermediate risk patients, respectively, until excellent response is obtained. Thereafter, chronic TSH suppression is not suggested in the majority of patients, and therapy is modulated to achieve low–normal TSH levels. As mentioned above, DTC patients have an overall good outcome, but the risk of persistent and recurrent disease remains not negligible (20–30%), requiring a protracted follow-up [5]. Thyroglobulin is the substrate for thyroid hormone biosynthesis and is released into the bloodstream in minimal amounts [7]. Then, “undetectable*” serum Tg is expected in patients who have obtained excellent response to treatment, while measurable Tg concentrations may indicate persistent or relapsing disease [8] (*when using ultrasensitive Tg assays whose limit of quantification is below 0.2 µg/L, Tg values at the lowest level can be measurable even after curative ablative therapy). Measurement of serum Tg and thyroglobulin autoantibodies (TgAb) is integral to promptly detecting persistent/recurrent DTC and re-stratifying the risk of disease recurrence and death [9].

The present paper briefly illustrates the laboratory issues, which may influence Tg and TgAb actionability in DTC patients, and focalizes in particular on the role of Tg and TgAb measurements in excluding/detecting DTC recurrences (i.e., diagnostic value), assisting in theranostic decisions (theranostic value) and stratifying the DTC patients’ prognosis (i.e., prognostic value), respectively.

## 2. Thyroglobulin: Biology and Laboratory Medicine

Thyroglobulin is a large and heterogeneous iodinated glycoprotein with 660 kDa, which is normally stored in the follicular colloid of the thyroid gland, being the substrate for the production of thyroid hormones [10]. Since Tg is produced only by well-differentiated thyroid cells, it is considered a reliable tumor marker for patients with DTC in the follow-up after total thyroidectomy and radioiodine ablation to monitor residual or recurrent disease [2]. Given that a measurable Tg concentration post-operatively suggests the presence of occult or overt residual disease, highly accurate and precise Tg measurement is crucial [9]. Serum Tg can be determined using immunoassays (radioimmunoassays and immunometric assays) and liquid chromatography–tandem mass spectrometry (LC-MS/MS) assays [10]. These methods have different analytical sensitivity, specificity and susceptibility to interference. Currently, most laboratories use immunometric assays to measure serum Tg (Tg-IMA), as they are available in high-throughput automated instruments with rapid turnaround times [11,12]. Current Tg-IMA has a very satisfactory ability to detect low serum Tg concentrations [13]. This characteristic is properly referred to as analytical sensitivity and includes three main analytical parameters: limit of detection (LOD), limit of quantitation (LOQ) and functional sensitivity (FS) (Table 3) [13,14,15,16,17].

The introduction of Tg-IMA with high analytical sensitivity into clinical practice has reduced the need to use thyroid hormone withdrawal or rhTSH stimulation to measure serum Tg concentrations during initial and long-term follow-up of patients with DTC [13]. Precisely, many studies have demonstrated that unstimulated serum Tg concentrations ≤ 0.2 μg/L ruled out additional stimulation tests in most cases [19,20,21,22,23]. Based on these observations, an expert consensus has reasonably defined Tg-IMA with LOQ ≤ 0.2 μg/L as “highly sensitive” [14] (Table 4).

As Tg is a large and heterogeneous iodinated glycoprotein, antibodies with different antigen specificities are used in Tg-IMA format [10]. Consequently, different Tg-IMA could provide different Tg concentrations in the same sample [11,24]. Although the introduction of the certified reference material (CRM 457)—now known as BCR^®^ 457—has reduced the variability between methods from approximately 60% to 30% [25], such difference remains significant, and the eventual variation in the method could cause an interpretative problem in serial monitoring of Tg [26,27]. In addition, the same Tg assay producer and, whenever possible, the same laboratory, should be maintained over the follow-up. If it is necessary to change the method, a period of overlap between the previous and the new entry assay should be performed to realign serum Tg levels [9,13], either prospectively or via parallel measurement of frozen reserve aliquots from previous follow-up examinations (if the institution carrying out the tumor follow-up has set up a reserve serum bank). Similar to other immunometric tests, the main limitation of Tg-IMA is their exposure to interferences, primarily heterophile antibodies (HAb), anti-Tg antibodies (TgAb) or substances such as biotin. HAb are human antibodies, which can bind to animal antigens [10]. In Tg-IMA, HAb can lead to a false-positive result (rarely a negative result) [28,29].

The presence of HAb results in formation of a bridge between the capture and detection antibody in the absence of the analyte [30]. HAbs affect approximately 1% of Tg measurements via Tg-IMA [31], although a recent paper by Barbesino et al. reported a higher HAb frequency of up to 3.6% [32]. Assessment of HAb interferences is not suggested as a routine practice and should be requested when discordances occur between measurable Tg concentrations and clinical presentation [30]. HAb interference can be assessed with various approaches, including [33] (1) use of an alternative immunoassay to measure Tg; (2) sample treatment with commercially available heterophilic blocking tubes; (3) verification of linearity following serial dilutions of the sample; (4) precipitation with polyethylene glycol (PEG); (5) use of LC-MS/MS to measure Tg (Tg-MS) [30,32]. TgAb interference must be considered when measuring Tg as a tumor marker [34]. Positive TgAb may be related to decreased or increased serum Tg concentrations in widely used Tg-IMA [35,36,37]. Approximately 20–25% of DTC patients have measurable TgAb levels at first diagnosis [9,38]. Consequently, in clinical practice, it is recommended to perform TgAb measurements together with serum Tg when monitoring patients with DTC in order to evaluate the reliability of the Tg result [2,14]. Two technical approaches have been described to determine the presence of TgAb: Tg recovery test, with its mini-recovery version, or measurement of serum TgAb concentration [37,39,40]. Currently, direct measurement of TgAb via immunoassays is more widely used, since recovery tests can only detect strong interferences, demonstrating no additional clinical benefit [2,14]. TgAb immunoassays were originally developed to diagnose autoimmune thyroiditis [38]. Although commercially available TgAb immunoassays claim standardization with respect to the first International Reference Preparation 65/93, several studies have demonstrated high variability in the analytical performance of the different TgAb immunoassays in terms of capability of detection (LOD, LOQ and FS), concordance between the methods and reference range and/or positivity cut-off for autoimmune thyroid disease [41,42,43,44] (Table 5).

Inter-method variability could be due to differences in the preparation and in the presentation of Tg in the assay, which could influence the exposure of immunodominant epitopes [11,42,45]. The poor agreement between methods has important clinical implications, as the classification and concentration of TgAb will differ depending on the assay [11]. Notably, the degree of interference is not related to serum TgAb levels, and it varies between patients and between Tg-IMA. Thus, any TgAb assay may not detect some interfering TgAb [9,34,42]. Since TgAb concentrations—still within the reference range and/or positivity cut-off—can already cause significant interference in Tg measurements, some experts have suggested the use of the method-specific LOD, LOQ or FS as a possible threshold for the detection of analytical interference of TgAb [13,14,27,41,46]. This analytical approach is not universally shared, and some other authors have proposed higher TgAb cut-offs derived from clinical data to reduce false-positive results. In fact, some study groups demonstrated that lower TgAb titers are unlikely to affect the clinical application of Tg as a tumor marker measured via Tg-IMA [47,48,49]. All in all, TgAb levels should be assessed using the same method over time and re-aligned when an assay change is unavoidable. This is relevant when TgAb levels are adopted as a surrogate tumor marker [14,50]. In recent years, the use of Tg-MS has been increasingly emerging to identify and overcome the interference of HAbs and TgAb in Tg measurement [33,51]. In fact, the use of trypsin digestion in the Tg-MS process results in the cleavage of all proteins, thus eliminating antibodies, which could constitute potential interferences [11,52,53]. Several studies reported an excellent agreement and correlation between Tg-MS and Tg-IMA, with the differences between them less than 10% in TgAb-negative cases [27,52,53]. Conversely, in most TgAb-positive cases, Tg concentrations are significantly higher in Tg-MS, although the degree of underestimation varies depending on the Tg-IMA used for comparison [33]. In a recent study, Barbesino et al. reported an excellent correlation between Tg-IMA and Tg-MS in both TgAb-negative samples (R^2^ = 0.94) and TgAb-positive samples (R^2^ = 0.86), with no statistically significant difference between the values obtained with the two methods in a cohort of subjects with DTC and positive for TgAb [32]. Notably, the results of this study demonstrated that, in patients with structural disease and positive for TgAb, serum Tg not detectable via Tg-IMA is often undetectable or, in any case, very low even if measured with Tg-MS, suggesting the absence of Tg in the majority of sera containing TgAb, both in patients with structural disease and in those free of disease [32]. Apart from the limited analytical sensitivity of TG-MS compared to the current Tg-IMA, other reasons have been hypothesized to explain the undetectable Tg via Tg-MS in patients with structural disease: the presence of a Tg variant, which alters the cleavage of trypsin sites, making the peptides obtained undetectable; the lack of Tg secretion by some DTCs despite being well-differentiated tumors; the increased metabolic clearance of the circulating Tg–TgAb complexes in TgAb-positive patients in vivo [10,32,54]. Further studies are necessary to support or not the proposed hypotheses [55]. Undoubtedly, there are still many steps to be taken toward the harmonization of Tg-MS assays. In particular, they still present substantial differences in the assignment of the calibrator, although the most recent methods show excellent concordance of the results [14,56]. Furthermore, Tg-MS remains an expensive and time-consuming technique, whose real clinical superiority in the measurement of Tg compared to Tg-IMA has not yet been demonstrated. For this reason, it is not a first-line test but only an alternative approach in selected cases [14]. Biotin may potentially interfere with many immunometric tests, resulting in falsely increased or decreased values [57]. Biotin is the hydro-soluble vitamin B7, which is present both in vegetables and meat [57]. This type of interference has emerged in recent years due to its wide use in the cosmetic field (up to 20 mg/day) and in the therapy of progressive multiple sclerosis at very high doses (up to 300 mg/day). Even Tg-IMA using streptavidin-biotin in their format may be susceptible to this type of interference, and being sandwich tests, the effect will be an underestimation, suggesting to the clinician an improvement in the patient’s disease status [58]. Little data are available on the interference of biotin in Tg measurement, and for the most part, these are clinical reports [57,59]. Currently, the extent of the interference depends on the dose taken, the digestion time and the Tg-IMA used [59]. If interference with biotin is suspected, it is recommended to re-sample after biotin termination for a period of time depending on the dosage taken [60]. Alternatively, it is recommended to consider using another method, which is not based on biotin-streptavidin binding, performing the serial dilution test and removing excess biotin using streptavidin-coated beads [61,62].

## 3. Thyroglobulin: Diagnostic and Prognostic Value

### 3.1. Pre-Operative Tg Measurement

For clarification of the malignant potential of thyroid nodules, Tg measurement is decidedly not recommended in the guidelines of international thyroid societies, such as the British Thyroid Association [63] or the American Thyroid Association [2]. The main argument for this is that an increased Tg release is found not only in thyroid follicular cell-derived carcinomas (DTC) but also in various benign thyroid processes (e.g., thyroid enlargement, benign thyroid nodules, hyperthyroidism, thyroiditis), and it is therefore very unspecific [64], in contrast to calcitonin, which has a relatively high specificity with regard to “screening” for *medullary* thyroid carcinoma (MTC) [65]. For example, in a case report, an extreme Tg elevation up to 22,000 ng/mL was recorded in a patient with Hashimoto’s thyroiditis [66]. However, there are constellations (“niche indications”) in which Tg measurement is discussed as a helpful diagnostic contribution. It is not always possible to exclude malignancy of thyroid lesions with the desired degree of certainty by using diagnostic imaging, and not all nodules that appear suspicious can be clarified using fine-needle aspiration cytology. This is particularly the case with multi-nodular goiters and with nodules in unfavorable locations. In addition, fine-needle biopsy results in Bethesda category I in up to 19% of cases and categories III and IV in up to 20% of cases [67,68,69]; since such cases predominantly turn out to be benign lesions, but there is still an increased risk of malignancy, there is generally a need for further clarification with the aim of improving the ratio of justified to avoidable surgical interventions. Since the availability of Tg assays, it has been investigated to what extent Tg measurement—in comparison or in addition to other diagnostic procedures—has a role in the differentiation between malignant and benign thyroid nodules [70]. Some of those studies focus on the Tg in cases where cytology is indeterminate. Following a systematic review evaluating publications from the period 2001–2014 [19], significant differences between benign and malignant nodules in the mean Tg values were described in most of the studies, and the Tg proved to be an independent predictor of malignancy; however, almost all studies found suboptimal accuracy in distinguishing malignant from benign nodules. Most of the evaluated studies used Tg cut-off values between 75 and 300 ng/mL, with the resulting sensitivity/specificity of 58–75%/49–76% (cut-off of 75 ng/mL) or 21–58%/62–95% (cut-off of 300 ng/mL). In some more recent studies, ROC analyses found folding sensitivities/specificities: 58%/91% at the optimal Tg cut-off of 188.5 ng/mL [71] and 72%/73% at 53 ng/mL [72]. When focusing on fine-needle-biopsied nodules with indeterminate cytology, DTC was confirmed in the majority (≥75%) of cases when this criterion was combined with the respective determined Tg cut-off value being exceeded, and in some studies, there was a significantly higher malignancy rate with increasing node size as an additional criterion [72,73,74,75]. Thus, elevated Tg values can serve as an argument to recommend surgery for nodules with indeterminate cytology. Some authors have suggested the inclusion of Tg measurement in the follow-up of patients who had to undergo irradiation of the neck region in childhood (mostly due to lymphoma) and therefore belong to a special risk group [76]. Another individual constellation is Tg measurement in cancer of unknown primary (CUP) syndrome when extensive metastases have been objectified in diagnostic imaging and an immunohistochemical classification is not available, especially if there is also a suspicious nodular thyroid finding [77,78,79]. Accordingly, in patients with CUP and thyroid nodules, DTC is present in most cases if the Tg value is significantly elevated (ROC analyses in order to determine a suitable cut-off value, however, were not carried out). Various attempts have been made to improve the specificity of the Tg measurement by linking it with other diagnostic parameters. For example, the value of a TSH/Tg ratio or Tg/TSH ratio was examined [80,81,82,83]. Ultimately, however, such ratio cannot significantly improve the accuracy of diagnosing a DTC.

### 3.2. Post-Operative Tg Measurement in Confirmed DTC

#### 3.2.1. Tg in Monitoring the Therapeutic Effect and Follow-Up Care

In contrast, Tg measured post-operatively in confirmed DTC has an undisputed central diagnostic role as a tumor marker [2,63], with its diagnostic specificity increasing the more radically the benign thyroid tissue had been ablated. The specificity of a single Tg value is maximum with additional radioiodine ablation of the remnant thyroid tissue, is usually still high with only surgical thyroidectomy and is—according to a recently published meta-analysis [84]—already limited if only lobectomy has been carried out. The release of Tg from both non-malignant thyrocytes and DTC cells is generally TSH-dependent, and the serum concentration under maximum TSH stimulation is on average an order of magnitude higher than under TSH-suppressive thyroid hormone administration. In a meta-analysis on the diagnostic value of Tg using first-generation assays [85], a diagnostic sensitivity of 0.778 ± 0.023 was determined for unstimulated Tg (under TSH-suppressive thyroid hormone medication), with a median cut-off of 2 ng/mL determined via ROC analysis; for stimulated Tg, on the other hand, the diagnostic sensitivity was 0.961 ± 0.013, with a median cut-off of 3 ng/mL. Accordingly, unstimulated Tg measurements performed with first-generation assays have insufficient diagnostic sensitivity. Therefore, Tg measurement under maximum TSH stimulation (6–18 months after total thyroidectomy and adjuvant radioiodine therapy) was long regarded as the gold standard for ensuring remission in patients without TgAb interference and was also recommended in many guidelines [2,63,86,87]. As far as the justification for this approach is concerned, all the above-mentioned guidelines mainly refer to two literature sources [88,89], according to which a 98–99.5% probability of a tumor-free status can be assumed if the TSH-stimulated Tg (s-Tg) value is below a defined cut-off (0.5 ng/mL in the BTA guideline, 1.0 ng/mL in the ATA guideline); in this case, further follow-up care may be limited to basal Tg measurements and high-resolution neck ultrasonography every 6–12 months. However, if the s-Tg value exceeds a defined cut-off (BTA: 1.0 ng/mL, ATA: 2.0 ng/mL), further diagnostic and therapeutic measures should follow. However, this concept also gave rise to criticism.

Achieving maximum TSH stimulation solely for the purpose of optimizing the diagnostic value of Tg measurement (without an indication for parallel radioiodine scintigraphy) seems inappropriate in terms of the burden on the patient (in the case of endogenous TSH stimulation) or the high costs of >1000 (in the case of exogenous stimulation with rhTSH).

The aforementioned guideline recommendations focus on a single s-Tg measurement 6–18 months after completion of primary therapy; although recurrences do indeed manifest themselves mostly within this early period, they can also occur many years later, which is why sensitive recurrence detection is also desirable in the further course.

The range of stimulability of Tg release achievable by TSH is very wide; less differentiated thyroid carcinomas are documented, which still have the capacity for basal Tg release but only have limited stimulability.

The proportion of DTC patients in whom the s-Tg is >2 ng/mL is given as 20–25% [87], whereby the Tg result of this patient population must be assessed as “false-positive” in as many as two-thirds of the cases, as they will remain free of clinical disease and will have stable or decreasing s-Tg levels over time.

The proportion of DTC patients in whom s-Tg values are measured in the gray zone (0.5–2.0 ng/mL) is reported to be 15–20% [87]. For this group of people, periodic repetitions of rhTSH-stimulated Tg measurement at approximately 1-year intervals are recommended until a pathological increase in Tg is no longer detectable; this is not a satisfactory solution in terms of patient burden, costs and efficiency.

In recent years, the treatment of DTC has become less radical for many tumor stages, which is why adjuvant radioiodine therapy is performed less frequently, and even total surgical thyroidectomy is more often avoided. Accordingly, the treated patients also come into follow-up care with a larger amount of Tg-secreting residual thyroid tissue. The aforementioned guideline recommendations are not designed for this situation.

The data underlying the guidelines are still based on the results of first-generation assays, and the question arises as to whether a change to these follow-up concepts is appropriate, given the highly sensitive Tg (hsTg) assays available since the new millennium.

Especially since almost all major suppliers of automated laboratory systems have been offering hsTg assays with a limit of quantification (LOQ) ≤ 0.2 ng/mL over the last 20 years, this technique has become increasingly established in laboratories, with a corresponding increase in the number of publications addressing the clinical evaluation of hsTg measurement. The focus of these publications was predominantly on the question of whether a single unstimulated hsTg (u-hsTG) value can predict the result of the s-Tg value—which was previously considered the gold standard for evaluating tumor status after thyroidectomy and RIT—with sufficient reliability. In a meta-analysis on this topic, in which the results of 3187 DTC patients (post-thyroidectomy, predominantly adjuvant RIT) from nine studies were evaluated, the predictive value of a u-hsTg of <0.1 ng/mL measured under L-thyroxine intake (with regard to exceeding a s-Tg cut-off of 1.0 or 2.0 ng/mL) was determined [19]. For the Access automated system used in most of the studies evaluated, an NPV of 97% (s-Tg cut-off of 1.0 ng/mL) or 99% (s-Tg cut-off of 2.0 ng/mL) and a PPV of 70% (s-Tg cut-off of 1.0 ng/mL) or 42% (s-Tg cut-off of 2.0 ng/mL) were calculated. Based on this, the authors conclude that s-Tg measurement in TgAb-free sera as a rule can be dispensed with if u-hsTg measurements are <0.1 ng/mL. However, in the meta-analysis cited above [19], the authors point out that the s-Tg value considered here as a reference is merely a surrogate for the tumor status and that the actual “gold standard” with regard to freedom from recurrence is a long-term clinical follow-up. To date, there are only a few studies, which have investigated the predictive value of u-hsTg measurement over a long-term follow-up. In a study where >10 years of follow-up were evaluated, there was an NPV/PPV with regard to recurrence-free survival for u-hsTg of 97.3%/35.7% (Tg cut-off of 0. 09 ng/mL) and 95.2%/85.7% (Tg cut-off of 0.2 ng/mL), respectively, and for s-Tg of 98.6%/36.4% (Tg cut-off of 0.5 ng/mL) and 96.7%/42.9% (Tg cut-off of 1.0 ng/mL), respectively. The authors conclude based on this that s-Tg and u-hsTg have a comparably high predictive power for recurrence-free survival [90]. The disadvantage of the limited specificity of a single u-hsTg value, which is only moderately above the LOQ, can be overcome by assessing the dynamics of the course of serial hsTg measurements under continuous thyroid hormone intake. As early as the 1980s, it was postulated that a continuous increase in Tg is highly suggestive of a progressive recurrence, even if no structural disease has yet been localized in the affected patient [91]. In the meantime, it has been proven that this also applies to the highly sensitive Tg range [92]. It is noteworthy that in all the guidelines cited at the beginning, the trend of serial Tg measurement under thyroid hormone intake in the course is classified as helpful for identifying recurrences and as superior to the single value, but none of these guidelines makes specific recommendations on how to approach this dynamic of Tg values quantitatively. Some studies have investigated the significance of the Tg doubling time (Tg-DT), analogous to the calculation of the doubling time of other tumor markers, such as calcitonin, CEA and PSA, which has been established for years. In a meta-analysis on this subject [93], a positive association was observed between Tg-DT < 1 year and recurrence or disease progression (for patients with Tg-DT < 1 year, the survival/risk ratio was 2.09). Furthermore, Tg-DT was found to be related with [^18^F]FDG PET/CT results among patients with negative radioiodine WBS. In a retrospective study [92], Tg-DT alone did not prove to be an independent survival predictor in all patients with progressive DTC (especially not in patients with only minor lymphogenic metastasis); however, when focusing on patients with a high tumor burden (Tg > 100 ng/mL), significant differences in survival rates were found when the Tg-DT was classified into the three groups of < 3 months, 3–12 months and >12 months. Concerning the Tg dynamics measured in the highly sensitive value range, the criterion “Tg increase of at least 0.1 ng/mL AND at least a doubling in the value compared to the Tg nadir measured during follow-up” seems to be a reasonable preliminary threshold for suspected recurrence, above which intensified localization diagnostic measures are justified [94]. Non-specific (“artificial”) continuous Tg increases—despite relatively stable corresponding TSH values—occur only rarely, for example with increasing development of heterophilic autoantibodies [31] or during pregnancy [95,96]. To investigate the gain of time, which could be achieved with serial u-hsTg measurements based on a usual follow-up cycle compared to a first-generation assay, hsTg re-determinations were carried out from frozen reserve samples from patients in whom recurrences were confirmed during follow-up from 6 months to 21 years after initial diagnosis of DTC [97]. At the corresponding time points, the Tg values measured with the first-generation assay were below its LOQ. With an approximately 6-month follow-up cycle, the recurrence was detectable 5–15 months earlier than with the first-generation assay due to a significant increase in hsTg values. Other authors [98] reported a similar gain of time of 6–12 months.

In a current expert consensus paper, detailed state-of-the-art recommendations for handling highly sensitive Tg measurements were published [14]. These recommendations state, among other aspects, that Tg should be measured after doctors—if possible—had waited at least 4 weeks following surgery and 4 months following RIT to assess Tg values and that TSH should always be measured simultaneously to determine TSH. The latter is relevant in demonstrating a comparable TSH stimulation in the follow-up period. Furthermore, it is stated that Tg measurement in particular with hsTg assays is not a reliable tool for disease recurrence in DTC patients after lobectomy. However, hsTg assays can be adopted in patients treated with total thyroidectomy who did not receive adjuvant RIT, as cured patients show low Tg levels, which will remain stable or even decline over the follow-up period [14].

#### 3.2.2. Tg in Post-Operative Decision Making: A Theranostic Marker?

Conventionally, post-operative DTC risk stratification integrates different prognostic clinical and pathological data obtained from pre-operative assessment, intra-operative findings and early post-operative testing, aiming to predict disease-specific mortality and risk of recurrence. Until 2015, most patients received radioiodine after surgery, and the role of Tg measurement was pivotal, as summarized above, in early detection of persistent or recurrent disease, the provision of prognostic information and long-term follow-up guidance for DTC patients. Then, a trend toward de-escalating iodine-131 (^131^I) treatment emerged in the following years [2]. A three-tiered post-operative risk evaluation based on histopathology is adopted to make decisions on post-operative ^131^I application. Inherently, such system cannot detect post-operative persistent disease (i.e., biochemical, structural or functional), which requires curative ^131^I administration. Notably, however, it should be noted that the decision between watchful waiting, remnant ^131^I ablation or adjuvant ^131^I therapy should be made depending on the individual tumor situation (i.e., confirmed or highly suspected persistent disease or not).

Therefore, the ATA risk groups remain important in patient management, but further variables should be taken into account, preferably in a multi-disciplinary tumor board, in line with the individualized, targeted therapeutic approach [99]. The post-operative administration of ^131^I, encompassing the diagnostic (i.e., post-therapy whole body scan, PT-WBS) and therapeutic dimensions, has been used for many years as the cornerstone for the detection of persistent disease, assessing ^131^I avidity and predicting ^131^I treatment response. However, the current omission of ^131^I treatment in many patients prevents the attending physician from obtaining that information, and other predictive markers are needed. In an effort to achieve an even more individualized therapeutic concept for each DTC patient after surgery, the importance of post-operative Tg determination before adopting further measures (“pre-ablation Tg” before adjuvant RIT) was recently investigated, as baseline pre-ablation Tg levels are robustly related to the chance of obtaining remission of disease or having persistent or recurrent disease following the initial ^131^I treatment [100]. Therefore, serum Tg was also proposed to inform ^131^I therapy in patients with low-risk DTC [101,102,103]. However, post-operative Tg is affected by several variables (i.e., the thyroid remnant tissue, the time passed following surgery, the Tg cut-off level, the TSH level and the risk of relapsing disease or metastases). Moreover, Tg autoantibodies (TgAb) may significantly affect the level of serum Tg in up to one-quarter of DTC patients early post-operatively [14]. Accordingly, Tg normal values mathematically normalized to serum TSH levels and thyroid remnant volume may enhance the reliability of post-operative serum Tg measurement, but technical problems still hamper this approach. Notably, early detection of post-operative persistent disease is pivotal to optimizing ^131^I treatment in terms of patients’ preparation and administered ^31^I activities to maximize the treatment’s effectiveness. Accordingly, identification of relapsing/metastatic DTC may cause an escalation in administered ^131^I activity. Recently, a retrospective multi-centric study was performed in a European population of 1317 DTC patients with negative TgAb, adopting a decision tree model to predict PT-WBS results [99]. This model integrated post-operative Tg and TSH levels, patients’ clinical and demographic data, thyroid remnant volume and ATA risk groups. It generated an algorithm predicting whether the DTC lesions following surgical treatment will be detected using PT-WBS. The combination of serum Tg values and lymph node involvement outperformed all other tested variables in predicting persistent disease after surgery and provided reliable support for making a clinical decision. Overall, the information provided is highly relevant to modifying the baseline risk stratification and selecting therapeutic rather than adjuvant ^131^I administration in patients with a high probability of persistent and/or metastatic disease.

## 4. Conclusions

The monitoring of DTC patients over time aims to timely detect the few individuals carrying post-treatment persistent or relapsing disease. Unstimulated hs Tg provides accurate diagnostic and prognostic information. More recently, adopting sophisticated statistical analysis (i.e., decision tree models), the use of Tg before radioiodine theranostic application proved to be useful in refining conventional, pathology-based risk stratification and informing a personalized application of additional adjuvant or therapeutic radioiodine administrations (i.e., radioiodine theranostics).

## Figures and Tables

**Table 1:** DTC recurrence risk classification (American Thyroid Association Criteria) [2].

Low-Risk DTC	Intermediate-Risk DTC	High-Risk DTC
No local or distant metastases	Microscopic ETE	Macroscopic ETE
All macroscopic tumor resected	cN1 or >5 pN1 (all < 30 mm)	Incomplete tumor resection
No extra-thyroid loco-regional invasion	Aggressive histology	Distant metastases
No aggressive histology or vascular invasion	Vascular invasion	Post-operative Tg levels consistent with distant metastases
131I given, no uptake outside the thyroid bed on PT-WBS	mPTC (m) with ETE and mutated BRAF V600E	High Tg levels compared to PT-WBS findings
cN0 or pN1 micro-MTS (<0.2 mm)	131I uptake outside the thyroid bed on PT-WBS	pN1 with any metastatic LN > 30 mm
Intra-thyroid FTC with capsular invasion and/or <4 foci of vascular invasion		FTC with >4 foci of vascular invasion

Legend: c, clinical; ETE, extra-thyroid extension; (m), multifocal; MTS, metastasis; N, lymph node; p, pathological; PT-WBS, post-treatment whole body scintigraphy; Tg, thyroglobulin.

**Table 2:** Different aims and characteristics of post-operative radioiodine administration.

	Aim	131I Activities	Preparation
Ablative	To eliminate thyroid remnant tissue and facilitate long-term follow-up	1.1–2.0 GBq	rhTSH preferred
Adjuvant	To lower the risk of recurrence	2.0–5.5 GBq	rhTSH preferred
Treatment	To treat persistent/recurrent disease	3.7–7.4 GBq	THW preferred

Legend: GBq, GigaBecquerel; rhTSH, recombinant human thyroid-stimulating hormone; THW, thyroid hormone withdrawal.

**Table 3:** Definition of the parameters defining analytical sensitivity [15,16,17,18].

Parameter	Definition
Functional Sensitivity (FS)	Concentration of thyroglobulin corresponding to a coefficient of variation of 20%. Determined in pools of thyroglobulin-autoantibody-negative patients in the clinically relevant concentration range, in two different lots of reagents and calibrators, and over a period of 6 months.
Limit of Detection (LOD)	The lowest analyte concentration distinguished from the limit of the blank with 95% confidence.
Limit of Quantitation (LOQ)	The lowest analyte concentration reliably measurable, within pre-defined accuracy goals for total allowable error (bias and imprecision). Determined with the use of 2 reagent lots, one instrument system, 3 days, at least 4 independent low-level samples and 3 replicates per day, resulting in at least 36 total low-concentration sample replicates per reagent lot (3 days × 4 independent low-level samples × 3 replicates).

**Table 4:** Main analytical characteristics of the most used thyroglobulin immunometric assays, as quoted by manufacturers [13].

Manufacturer	Tg Assay	Principle	Analytical Sensitivity (µg/L)	Assay Classification
Abbott	Architect Tg	CLIA	LOB 0.05	High sensitivity
			LOD 0.09	
			LOQ 0.14	
Abbott	Alinity I Tg	CLIA	LOB 0.07	High sensitivity
			LOD 0.09	
			LOQ 0.14	
Beckman Coulter	Access Tg	CLIA	AS ≤ 0.1	High sensitivity
BRAHMS Thermofisher	BRAHMS h-Tg Sensitive KRYPTOR	TRACE	LoD 0.09	High sensitivity
			LoQ 0.17	
			FS 0.15	
Diasorin	Liaison^®^ Tg II Gen	CLIA	LOD 0.1	High sensitivity
			LOQ 0.17	
Mindray	Thyroglobulin (Tg)	CLIA	AS ≤ 0.1	High sensitivity
Roche Diagnostics AG	Elecsys Tg II	ECLIA	LOB 0.02	High sensitivity
			LOD 0.04	
			LOQ 0.1	
Siemens Healthineers	Atellica^®^ IM	CLIA	LOB 0.026	High sensitivity
			LOD 0.036	
			LOQ 0.05	
Siemens Healthineers	Immulite 2000 Tg	CLIA	LOD 0.2	Conventional
			FS 0.9	
Shenzhen New Industries Biomedical Engineering Diagnostic	Maglumi^®^ TG	CLIA	LOB 0.1	Conventional
			LOD 0.25	
			LOQ 0.8	

Legend: CLIA, chemiluminescent assay; CV, coefficient of variation; ECLIA, electro-chemiluminescence assay; AS, analytical sensitivity; FS, functional sensitivity; LOB, limit of blank; LOD, limit of detection; LOQ, limit of quantitation; M, manufacturer; Tg, thyroglobulin; TRACE, time-resolved amplified cryptate emission. Note: Tg assays with functional sensitivity or LOQ higher than 0.2 µg/L are classified as conventional; Tg assays with functional sensitivity or LOQ of 0.2 µg/L or less are referred to as high sensitivity. Information updated in January 2024.

**Table 5:** Main analytical characteristics of the most used antithyroglobulin antibody immunoassays, as quoted by manufacturers [38,41,42,43].

Manufacturer	TgAb Assay	Principle	Analytical Sensitivity (kIU/L)	MCO (kIU/L)
Abbott Diagnostics	ARCHITECT Anti-Tg	CLIA	LOD 0.07	4.11
			FS 0.31	
Abbott Diagnostics	Alinity I Anti-Tg	CLIA	LOB 0.05	4.11
			LOD 0.11	
			LOQ 0.33	
Beckman Coulter	Access Thyroglobulin Antibody II	CLIA	LOB 0.17	4
			LOD 0.37	
BRAHMS Thermofisher ^a^	BRAHMS ANTI-TGn KRYPTOR	TRACE	LOD 9	33
			LOQ 42.4	
			FS 33	
Diasorin	LIAISON^®^ Anti-Tg	CLIA	LOD 5	100
			LOQ 10	
Mindray	Antibody to thyroglobulin (anti-TG)	CLIA	AS ≤ 0.9	4
Roche Diagnostics	Elecsys Anti-Tg	ECLIA	LOB 7	115
			LOD 10	
			LOQ 15	
Siemens Healthineers	Atellica^®^ IM Anti-Thyroglobulin II (aTgII)	CLIA	LOB 0.7	1.3 ^b^ 4.5 ^c^
			LOD 0.9	
			LOQ 0.9	
Siemens Healthineers	IMMULITE^®^ 2000 Anti-TG Ab	CLIA	LOD 2.2	40
Shenzhen New Industries Biomedical Engineering Diagnostic	Maglumi^®^ TGA	CLIA	LOD 0.5	95

Legend: AS, analytical sensitivity; CLIA, chemiluminescence immunoassay; ECLIA, electro-chemiluminescence immunoassay; FS, functional sensitivity; LOD, limit of detection; LOQ, limit of quantitation; MCO, manufacturer cut-off level; TgAb, antithyroglobulin antibodies; TRACE, time-resolved amplified cryptate emission. ^a^ All methods are standardized with the International Reference Preparation 65/93 and use International Units (kIU/L), except for BRAHMS ANTI-TGn, which use kAU/L. Information updated on January 2024. ^b^ Obtained from apparently healthy subjects. ^c^ Suggestive of autoimmune thyroid disease.

## Data Availability

Not applicable.
